# Soybean-Enriched Snacks Based on African Rice

**DOI:** 10.3390/foods5020038

**Published:** 2016-05-20

**Authors:** Mauro Marengo, Hannah F. Akoto, Miriam Zanoletti, Aristodemo Carpen, Simona Buratti, Simona Benedetti, Alberto Barbiroli, Paa-Nii T. Johnson, Esther O. Sakyi-Dawson, Firibu K. Saalia, Francesco Bonomi, Maria Ambrogina Pagani, John Manful, Stefania Iametti

**Affiliations:** 1 Department of Food, Environmental and Nutritional Sciences (DeFENS), University of Milan, Milan 20133, Italy; mauro.marengo@unimi.it (M.M.); miriam.zanoletti@unimi.it (M.Z.); aristodemo.carpen@unimi.it (A.C.); susanna.buratti@unimi.it (S.B.); simona.benedetti@unimi.it (S.B.); alberto.barbiroli@unimi.it (A.B.); francesco.bonomi@unimi.it (F.B.); ambrogina.pagani@unimi.it (M.A.P.); 2 Department of Nutrition and Food Science, University of Ghana, Legon P.O. Box LG 25, Ghana; nobleakoto@gmail.com (H.F.A.); esakyid@ug.edu.gh (E.O.S.-D.); fsaalia@ug.edu.gh (F.K.S.); 3 Council for Scientific and Industrial Research (CSIR)—Head Office, Accra P.O. Box M32, Ghana; paanii.johnson@gmail.com; 4 Africa Rice Center, Cotonou 01 B.P. 2031, Benin; J.Manful@cgiar.org

**Keywords:** e-nose, e-tongue, extrudates, rice, soybean enriched snacks

## Abstract

Snacks were produced by extruding blends of partially-defatted soybean flour with flours from milled or parboiled African-grown rice. The interplay between composition and processing in producing snacks with a satisfactory sensory profile was addressed by e-sensing, and by molecular and rheological approaches. Soybean proteins play a main role in defining the properties of the protein network in the products. At the same content in soybean flour, use of parboiled rice flour increases the snack’s hardness. Electronic nose and electronic tongue discriminated samples containing a higher amount of soybean flour from those with a lower soybean flour content.

## 1. Introduction

Changes in eating patterns in West Africa are occurring at an increasing rate due to urbanization, globalization, economic, and demographic trends. This is further fueled by changes in the social structure as a result of the increase in the number of mothers working outside their home and in the increasing demand for convenience foods [1]. These changes in eating patterns include an increasing consumption of snacks (cookies, nuts, extruded snacks) in all age groups. Snacks provide a significant part of the nutrient and calorie intake for many African consumers [2]. Currently, high snack consumption in Ghana is associated to their widespread presence in open markets, supermarkets, petty trading, and restaurants in both urban and rural areas [3]. Extrusion-cooking has found widespread application in the cereal-based snack food industry, because of its ease of operation and of its ability to produce a number of consumer-appealing textures and shapes [2]. Though wheat- and corn-based snacks are the most popular products in Ghana, rice flour has become an attractive ingredient for the production of extruded snacks due to its bland taste, hypoallergenicity, availability, and high digestibility [4].

Rice has become a staple in most West African countries [5], including both rural and urban areas [6,7]. The strong increase in the local rice production is yet not sufficient to meet the increasing demand. In addition, African consumers prefer imported rice to locally-produced rice, due to low grain quality, low head yield, high chalkiness, poor cooking performance, and taste of locally-grown rice [8]. In this frame, the Africa Rice Center (AfricaRice, Cotonou, Benin) and the Global Rice Science Partnership (GRiSP) are pioneering programs aimed at the development of new added-value products based on flour from local rice. These products include pasta, baby food, puddings, and extruded snacks. Since rice has a relatively low protein content, the development of nutritious snacks (*i.e.*, being valuable sources of protein and energy) requires the combination of rice with other protein sources. In this frame, soybean is an excellent and relatively inexpensive source of proteins [9]. Blending of soybean with rice should provide a good and well-balanced protein intake, along with other functional, nutritional, and health-related beneficial effects [10].

Therefore, this study aimed at developing soybean-enriched rice-based extruded snacks, and at addressing their properties by a combination of physical, molecular, and instrumental sensory approaches.

## 2. Experimental Section

### 2.1. Rice and Soybeans

A locally-grown African rice variety (Togo Marshall), was provided by the Ghana Rice Inter-Professional Body (GRIB, Accra, Ghana). Parboiling, when required, was carried out at the Food Research Institute of the Council for Scientific and Industrial Research (CSIR-FRI, Accra, Ghana), and milling was performed at a local rice mill. Rice flour with a particle size distribution centered at 500 μm was obtained from either raw or parboiled rice.

Soybean (Nangbaar variety) was provided by Crops Research Institute of the CSIR, Ghana. Seeds were cleaned, dried in an oven at 50 °C for 45 min, and dehulled by using a disc attrition mill. Cotyledons were separated from the coats by winnowing, partially defatted via a screw press, and milled into flour to a particle size distribution centered at 850 μm.

### 2.2. Production of Soybean-Enriched Extruded Rice Snacks

Various formulations were prepared in several (*n* ≥ 6) replicate batches by using flour from either untreated rice (U) or parboiled rice (P), and 10% or 25% (*w/w*) of partially-defatted soybean flour. These formulations represent the ones with high preference of local consumers for rice-based products, as reported in other studies on rice products in the African market [3]. Formulations containing more than 25% partially-defatted soybean flour scored very low with the panel as for mouth feel and aftertaste, as reported for other soybean-enriched foods [11].

Each of the four formulations (labeled as U-10, U-25; P-10, P-25) was extruded through a circular die (4 mm diameter) by an intermeshing co-rotating twin screw extruder (CLEXTRAL BC 21, Firminy, France) into 4 cm long pellets, by using the same conditions: constant screw speed 1000 rpm; barrel temperature 200 °C; and feed moisture 25%.

### 2.3. Protein Characterization

The nature of interactions stabilizing protein aggregates in the extruded products was addressed by measuring soluble proteins from extruded products in various buffers as described in [12]. Proteins were extracted in triplicate by dispersing 0.15 g of finely ground samples (<250 μm) in 5 mL of 0.05 M sodium phosphate buffer, pH 7.0, containing 0.1 M NaCl. After stirring at room temperature for 60 min, suspensions were centrifuged at 10,000× *g* for 20 min at 20 °C, and the protein content in the supernatant was assessed by a dye-binding method [13]. Where indicated, protein extraction was carried out as above but in the presence of 6 M urea or of 6 M urea and 10 mM dithiothreitol (DTT).

SDS-PAGE was performed according to [14]. A volume of the proteins solutions resulting from solubilization in the various buffers was treated with an equal volume of denaturing buffer (0.125 M Tris-HCl, pH 6.8, 50% glycerol, 1.7% SDS; 1% 2-mercaptoethanol; 0.01% Bromophenol Blue). Samples were boiled for 10 min, and volumes corresponding to 0.015 mg protein were loaded onto a fixed porosity gel (12% monomer). SDS-PAGE was carried out in a MiniProtean apparatus (Bio-Rad, Richmond, VA, USA), and gels were stained with Coomassie Brilliant Blue. Molecular mass markers covered the range between 14 and 96 kDa.

Accessible thiol groups (expressed as µmol thiols (g sample)^−1^) were determined in triplicate as described in [15], with slight modifications. A 0.15 g aliquot of a finely ground sample was suspended in 5 mL of 0.05 M sodium phosphate, 0.1 M NaCl, pH 7.0, 0.2 mM 5,5′-dithiobis-(2-nitrobenzoate) (DTNB), in the presence or in the absence of 6 M urea as indicated. After 60 min stirring at 25 °C, the suspension was centrifuged (10,000× *g*, 20 min, 15 °C) and the absorbance of the supernatant was read at 412 nm against a proper blank.

### 2.4. Physical Properties of the Products

A portable colorimeter CR-300 (Minolta, Osaka, Japan) was used to assess the color of extruded products in terms of L* (lightness), a* (redness), and b* (yellowness) values in the CIELAB color space [16]. L*, a*, and b* were measured directly on finely ground samples (<250 μm) homogenously distributed in a Petri dish. The values reported in Table 1 are the average of 16 independent measurements.

Texture analysis on the extruded products was carried out on samples punctured by using a Zwick Z005 testing machine equipped with a 100 N load cell (Zwick GmbH and Co., Ulm, Germany) fitted with a 4-mm diameter cylindrical flat-faced probe, at a test speed of 1 mm/s. Twenty measurements were carried out for each product and force-deformation curves were recorded until 50% of sample penetration. Work values (*N* × mm) were normalized to the sample diameter (mm). Expansion ratios were calculated as the ratio between the average sample diameter (mm) and the extruder circular die diameter (mm).

### 2.5. E-Sensing: Electronic Nose and Electronic Tongue

Volatile profile analyses were performed by using a Portable Electronic Nose (PEN2, Win Muster Airsense Analytics Inc., Schwerin, Germany). It consists of a sampling apparatus, a detecting unit containing the sensor array, and an appropriate pattern-recognition software (Win Muster version 1.6, Airsense Analytics Inc., Schwerin, Germany) for data recording and elaboration. For electronic nose (e-nose) measurements, the sample headspace is exposed to sensors and the interaction between sample and sensors provides a signal pattern that depends on the sensors selectivity and sensitivity to volatile compounds in the sample headspace [17]. The sensor array is composed of 10 metal oxide semiconductor (MOS) type sensors: W1C (aromatic); W5S (broadrange); W3C (aromatics); W6S (hydrogen); W5C (aromatics-aliphatics); W1S (broad-methane); W1W (sulphur-containing compounds); W2S (broad-alcohol); W2W (sulphur-containing and chlorinated compounds); W3S (methane-aliphatics). For the determination of the aroma profiles of the extruded products, 0.2 g of sample was placed in a 40 mL airtight glass vial fitted with a pierceable silicon/Teflon^®^ disk in the cap. After 1 h equilibration at room temperature, headspace measurements were performed according to the following conditions: flow rate 300 mL·min^−1^, injection time 60 min, flush time 180 min (during which the surface of the sensors was cleaned with air filtered through active carbon). All samples were analyzed twice and the sensor response averages were used for subsequent statistical analysis.

Electronic tongue (e-tongue) measurements were performed by a Taste-Sensing System SA 402B (Intelligent Sensor Technology Co. Ltd., Atsugi, Japan). For this study a total of five detecting sensors and two reference electrodes were used, separated in two arrays according to membrane charge: hybrid (CT0; CA0; AAE) and positive (C00; AE1). Measurements are based on the capability of tasty compounds to modify sensor potential through electrostatic or hydrophobic interactions [18]. Extruded products were milled into fine flour, of which 3 g were suspended into 30 mL of distilled water. Suspensions were vortexed for 2 min and centrifuged at 5000× *g* for 5 min at room temperature. Supernatants were filtered through a 0.45 μm filter (Millipore, Vimodrone, Italy) and diluted 1:4 (*w/w*) with distilled water to get the solution to be tested. Prior to the measurement, the detecting sensors and reference electrodes were dipped into a reference solution (30 mM potassium chloride, 0.3 mM tartaric acid) and the electric potential was measured for each sensor (Vr). The sensors were then dipped for 30 s into the solution obtained from the extruded products, and a potential (Vs) was measured. For each sensor the “relative value” (Rv) was calculated as the difference between the potential of the sample and that of the reference solution (Vs-Vr). Sensors were rinsed with fresh reference solution for 6 s and then dipped into the reference solution again. The new potential of the reference solution was defined as Vr’. For each sensor, the difference between the potential of the reference solution before and after sample measurement (Vr’-Vr) is the CPA (change of membrane potential caused by absorption) value (CPAv) and corresponds to the e-tongue “aftertastes”. Before a new measurement cycle started, electrodes were rinsed for 90 s with water and then for 180 s with the reference solution. Each sample was evaluated in duplicate and sensor output were converted to taste information. 

The “taste values” were calculated by multiplying sensor outputs for appropriate coefficients based on the Weber-Fechner law, which gives the intensity of sensation considering the sensor property for tastes [19]. In particular, the “taste values” were estimated as:

(1) Sourness = 0.3316 × Rv(CA0)

(2) Saltiness = −0.252 × Rv(CT0)

(3) Bitterness = −0.140 × Rv(C00) + 0.084 × Rv(CT0)

(4) Aftertaste-bitterness = −0.210 × CPAv(C00)

(5) Astringency = 0.1575 × Rv(AE1) + 0.1575 × Rv(CT0)

(6) Aftertaste-astringency = −0.252 × CPAv(AE1)

### 2.6. Statistical Analysis

E-nose and e-tongue data were elaborated by using the MINITAB 14, version 12.0 software (Minitab Inc., State College, PA, USA) package. Principal Component Analysis (PCA) was applied as an exploratory tool to uncover aroma and taste characteristics. For all other measurements, ANOVA was performed on the data adopting the least significant difference (LSD). Data were processed by Statgraphic Plus for Windows version 5.1. (StatPoint Inc., Warrenton, VA, USA).

## 3. Results and Discussion

### 3.1. Overall Protein Organization

Structural features of proteins in soybean-enriched rice-based extruded snacks were evaluated by extracting proteins in buffers with different dissociating ability towards covalent and non-covalent inter-protein bonds. This approach provides useful information on the nature of bonds that stabilize aggregation and/or association in protein-based polymers [12,14,15].

**Figure 1:**
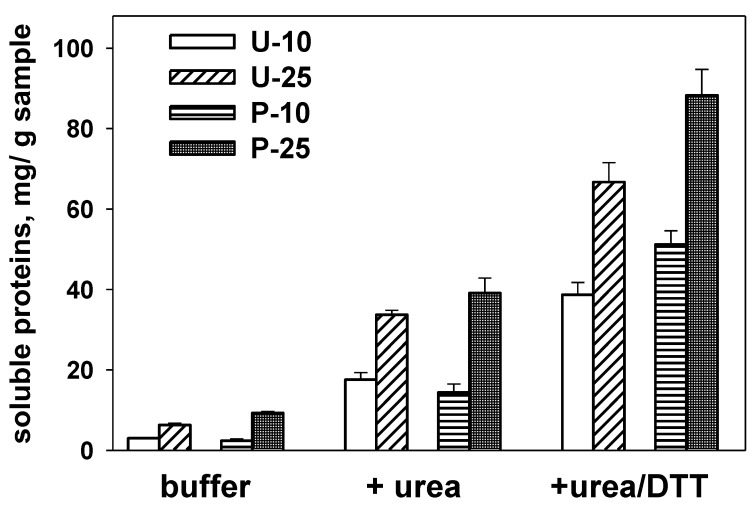
The amount of proteins solubilized from the various samples in different buffer systems. Aliquots of the various samples were suspended under stirring in 0.05 M sodium phosphate, 0.1 M NaCl, pH 7.0, in the presence/absence of 6 M urea and 10 mM DTT, as indicated. Different letters for results obtained with each buffer system indicate significant differences (*p* ≤ 0.05). Samples are identified by letters (indicating the use of untreated (U) or parboiled (P) rice) and digits (10, 25) indicating the percent content in soybean flour.

As shown in Figure 1, the amount of proteins soluble in buffered saline (*i.e.*, albumins and globulins) is very low and comparable in all snacks. Addition of a denaturing agent (urea) to the extraction buffer results in an increase of the amount of soluble proteins, due to dissociation of aggregates stabilized by hydrophobic interactions [20]. A further increase in solubilized proteins was evident when both urea and the disulfide-reducing agent dithiothreitol were present. In these conditions, disulfide linkages are reduced, allowing the destabilization of aggregates involving proteins of different origin, as previously reported for other cereal-based enriched foods [21,22]. The data in Figure 1 makes it evident that the amount of proteins solubilized from snacks prepared with parboiled rice (P-10 and P-25) is higher—under otherwise comparable conditions as for the extractant and soybean flour content—than what is obtained from those prepared with milled rice (U-10 and U-25).

All together, these results suggest the presence in all extruded snacks of a structured protein network stabilized by both hydrophobic interactions and inter-protein disulfide bonds, although each product shows peculiar formulation-related features. Indeed, the amount of soluble proteins in all buffers depends on both the soybean flour content and on the parboiling of rice prior to the extrusion process. A higher amount of soluble proteins was detected with increasing soybean flour content. When comparing products with the same soybean flour content, the amount of soluble proteins was higher when samples were prepared with parboiled rice. This may suggest that previously parboiling of rice prevents some of the interactions between rice and soybean proteins taking place during the extrusion process, thus affecting the properties of the protein network in the final product.

**Figure 2:**
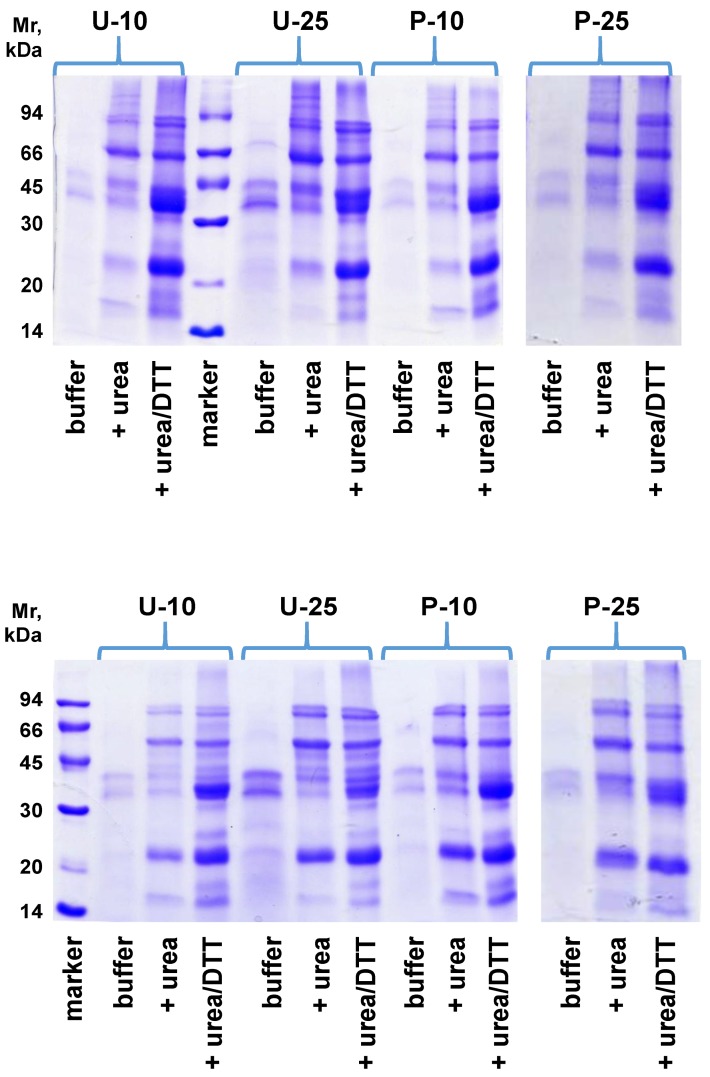
SDS-PAGE profile of proteins solubilized from the various samples in different buffer systems. Aliquots of the various samples were suspended under stirring in 0.05 M sodium phosphate, 0.1 M NaCl, pH 7.0, in the presence/absence of 6 M urea and 10 mM DTT, as indicated. Separations were run on protein samples denatured in the absence (top) or in the presence (bottom) of 2-mercaptoethanol. Samples are identified by letters (indicating the use of untreated (U) or parboiled (P) rice) and digits (10, 25) indicating the percent content in soybean flour.

Proteins solubilized from the extruded snacks in the various buffer systems used in the studies presented in Figure 1 were separated by SDS-PAGE, thus allowing a comparison among samples in terms of representative protein families and an estimation of their size. The results of SDS-PAGE carried out in the absence/presence of 2-mercaptoethanol—a disulfide reducing agent—are presented in Figure 2. As expected from the conditional solubility results, the number and the intensity of bands increase when urea or urea/DTT are used for protein extraction, confirming the presence of protein aggregates stabilized by hydrophobic interactions and disulfide bonds. However, the tracings obtained for extrudates containing various levels of soybean flour and flour from differently treated rice do not offer evidence for the involvement of specific polypeptides and proteins in the aggregation events made evident by the conditional solubility studies (see above). In other words, the differences related to formulation or pre-treatment are not associated with a particular protein.

Thiol-disulfide exchange reactions are among the major contributors to the formation of a covalently-linked protein network in many foods, where disulfides represent the most “natural” type of inter-protein covalent bond [14]. In this frame, it may be pointed out that thiol-disulfide exchange reactions occur as a function of the accessibility of the involved thiols which, in turn, depends on structural features of the proteins.

The degree of structural “stiffness” of the protein network in individual samples was evaluated through thiol accessibility studies in the presence/absence of urea. These studies were carried out on protein suspensions [12,23], and the measurements provide two separate parameters, namely, the total content in readily accessible thiols (measured under non-denaturing conditions on both the soluble and insoluble fraction) and the increment in thiol accessibility due either to urea-induced protein unfolding and/or to urea-dependent breakdown of hydrophobic interactions among aggregated proteins.

**Figure 3:**
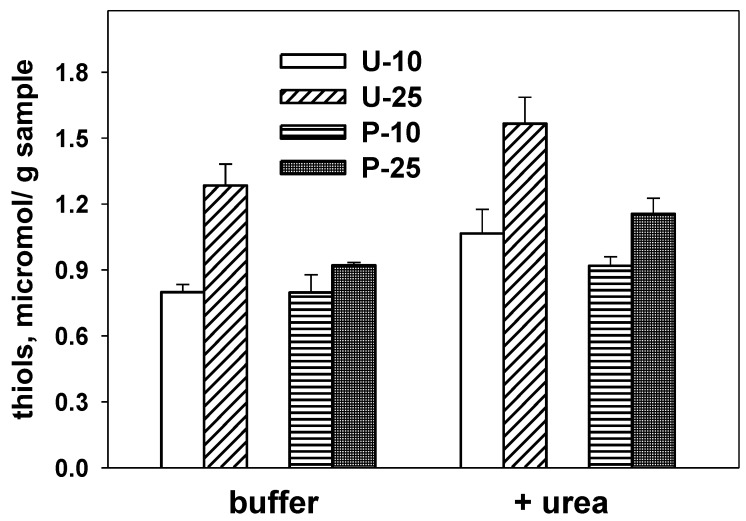
Accessible thiols content of proteins in the various products. Thiols were assessed on finely-ground samples suspended in 0.05 M sodium phosphate, 0.1 M NaCl, pH 7.0, in the presence/absence of 6 M urea as indicated. Different letters for results obtained with each buffer system indicate significant differences (*p* ≤ 0.05). Samples are identified by letters (indicating the use of untreated (U) or parboiled (P) rice) and digits (10, 25) indicating the percent content in soybean flour.

Results in Figure 3 show that the amount of detectable thiols in all the extruded products is low, and increases only slightly in the presence of a denaturing agent. The amount of accessible thiols increases with the soybean flour content, and is significantly higher—at least at high soybean levels—in extruded snacks prepared from untreated rice than in samples based on parboiled rice. This suggests that reticulation of soybean proteins to give a sturdy protein network (with limited accessibility of the protein thiols) is somewhat occurring to a larger extent when using parboiled rice.

### 3.2. Physical Properties

Color is one of the most important quality factors directly related to the acceptability of food products [24]. In extruded-cooked products, color analysis can provide hints about the extent of occurrence of Maillard-type reactions, as well as on the degree of pigment degradation occurring during the extrusion process.

As shown in Table 1, a decrease in lightness (L*), and an increase in redness (a*) and yellowness (b*), was detected as the soybean flour content increased in snacks produced from either untreated or parboiled rice. This is due to the increased protein content of the formulations, which favors Maillard-type browning reactions in carbohydrate-rich matrices, as described in a number of studies [25,26]. Snacks prepared with parboiled rice flour show lower lightness (L*) values and higher redness (a*) and yellowness (b*) parameters than samples from milled rice flour. This results from a darker color of the parboiled rice, which depends on the heat-related browning events and on the diffusion of husk pigments into the endosperm during the parboiling treatment.

The textural properties of snack were investigated. Hardness and expansion ratio are fundamental quality parameters of extruded snacks, that depend on the size and number of gas bubbles within the snack rigid matrix, and may be conveniently addressed by using a dynamometer [27,28]. Additionally, as shown in Table 1, the puncture force significantly increases with increasing soybean flour content, favoring formation of a compact protein network.

The rigid network formed at high protein content may affect product expansion, as well as changes in the structure of starch [27]. However, we confirm that—as reported in many studies—starch was completely gelatinized in all extrudates, as indicated by thermal analysis and pasting properties measurements (not shown). Thus, product expansion is governed mostly by the protein component, and is expected to be inversely related to the rigidity of the protein network. Accordingly, the rigid protein network consequent to the parboiling treatment resulted in increased sample hardness. In other words, the parboiled rice seems to be unable to develop a matrix that entraps the water vapor and forms bubbles.

### 3.3. E-Sensing

**Figure 4:**
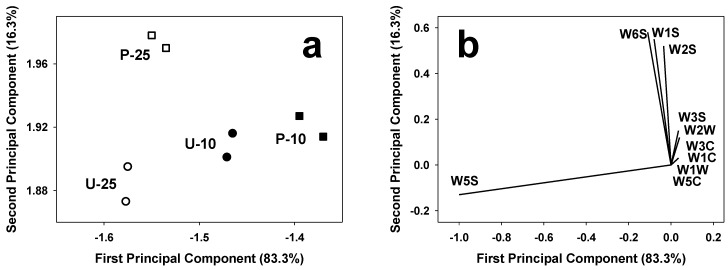
E-nose PCA score plot (**a**) and loading plot (**b**). Samples are identified by letters (indicating the use of untreated (U) or parboiled (P) rice) and digits (10, 25) indicating the percent content in soybean flour.

E-sensing investigation of the selected extruded products was initially performed by using the e-nose for the assessment of the volatiles profile. E-nose data were elaborated by PCA in a covariance matrix, and the two first principal components accounted for 99.6% of the total variance (Figure 4). The score plot (Figure 4a) allows the discrimination of the extruded samples on the basis of the rice treatment prior to extrusion, and on the amount of soybean in the formulation. Samples are distributed on the first principal component (PC1) from the right to the left according to the soybean content. In particular, products with a low soybean content are located on the right of the plot, whereas those with high soybean content are placed to the left. With respect to rice treatment, milled rice samples are clustered in the lower part of the second principal component (PC2), and parboiled rice samples are located in the upper part of PC2.

By considering the loading plot (Figure 4b), it is possible to notice that one sensor of broad range sensitivity (W5S) is relevant in the discrimination of samples on the PC1 according to their soybean content. On the PC2, sensors of the WS series (W6S, W1S, and W2S, of broad sensitivity and sensitive to alcohol and methane) were able to discriminate samples on the basis of rice treatment.

The influence of a different soybean content on the electronic nose responses may result from the different lipid content of the final products which might affect snacks aroma profile (and any possible off-odors caused by lipid oxidation) during their shelf-life [29]. Reportedly, rice parboiling results in starch gelatinization and in transfer of volatiles from the rice husks to the rice endosperm. This may have a strong influence on the aroma of the resulting products, and may explain the similar behavior of samples from parboiled rice on PC2 [30].

**Figure 5:**
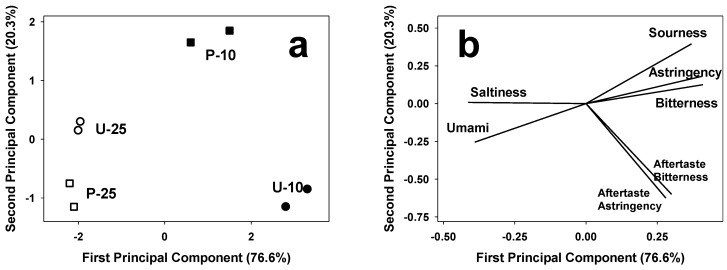
E-tongue PCA score plot (**a**) and loading plot (**b**). Samples are identified by letters (indicating the use of untreated (U) or parboiled (P) rice) and digits (10, 25) indicating the percent content in soybean flour.

The e-sensing investigation was completed by e-tongue measurements. An e-tongue is a liquid analytical device that mimics the taste-sensing mechanism of the gustatory system and comprises sensor arrays specific for liquid samples. With respect to consumer acceptability and compliance to quality standards, taste is one of the major factors determining the market penetration and commercial success of food products such as snacks [31]. The taste values collected by e-tongue were analyzed by PCA in correlation matrix, and the two first principal components accounted for 96.9% of the total variation in the taste of the extruded samples under investigation (Figure 5). Considering the score plot (Figure 5a), extruded snacks are discriminated on PC1 (accounted for 76.6% of the total variance) on the basis of the soybean content in the formulation, regardless of rice treatment. Snacks with a high soybean content clustered to the left (negative) side of PC1, whereas those with low soybean content were located in the right side of the plot. The loading plot (Figure 5b) shows that a high soybean content results in umami and salty tastes, whereas products with a low soybean content tasted bitter, sour, and astringent, as reported in studies, where soybean flours or isolates were used to fortify bread [11] and a number of other food products [32].

## 4. Conclusions

The combination of biochemical and physical approaches has allowed the authors to address the molecular properties of soybean-enriched rice-based extruded snacks and to correlate them with products’ macrostructures. Both the extent of soybean addition and the rice parboiling before extrusion play a key role in defining the molecular, textural and sensory properties of the final products.

In particular, the soybean levels affect sensory traits (as detected by e-nose and e-tongue), whereas the use of parboiled rice affects the network-forming ability of the proteins in the system. As a consequence, at a given soybean content, snacks prepared from parboiled rice are harder and less expanded than those from untreated rice.

This information can offer some guidelines with regards to designing and producing snacks conforming to the local consumers’ expectations. Hopefully, the approaches and the data presented here about the significance of interactions among nutritionally complementary ingredients may be used to add value to Africa-grown raw materials.

## Figures and Tables

**Table 1:** Physical characteristics of extruded snacks.

Sample	Color				
	L*	a*	b*	Hardness, *N*	Expansion Ratio
U-10	87.37 ± 0.51 ^a^	−0.63 ± 0.06 ^a^	15.14 ± 0.24 ^a^	3.8 ± 0.74 ^a^	3.6 ± 0.14 ^a^
U-25	84.10 ± 0.21 ^b^	0.11 ± 0.02 ^b^	19.72 ± 0.29 ^b^	5.9 ± 0.83 ^b^	3.3 ± 0.24 ^b^
P-10	82.61 ± 0.56 ^c^	0.31 ± 0.06 ^c^	19.31 ± 0.35 ^b^	5.9 ± 0.62 ^b^	3.1 ± 0.34 ^b^
P-25	81.74 ± 0.92 ^d^	0.62 ± 0.11 ^d^	21.25 ± 0.63 ^c^	8.8 ± 0.92 ^c^	2.4 ± 0.44 ^c^

Values are means ± standard deviations (*n* = 20 for physical measurements, *n* = 16 for color measurements). Different letters in the same column indicate significant differences (*p* ≤ 0.05). Samples are identified by letters (indicating the use of untreated (U) or parboiled (P) rice) and digits (10, 25) indicating the percent content in soybean flour.
